# Proceedings of the Buffalo Medical and Surgical Association

**Published:** 1888-10

**Authors:** W. H. Bergtold

**Affiliations:** Secretary


### PROCEEDINGS OF THE BUFFALO MEDICAL AND SURGICAL ASSOCIATION.

Reported by W. H. BERGTOLD, M. D., Secretary.

Regular monthly meeting, held September 4, 1888, in the Buffalo Library Building; President VAN PEYMA in the chair.

Minutes read and approved.

DR. EDWARD CLARK then read on "The Prevalence of Small-Pox and its Management." (See page 97, this number.) He also exhibited several photographs of patients taken while they were in the Quarantine Hospital. Dr. Clark took the photographs himself, having been unable to get a photographer to do it at a reasonable price.

DISCUSSION:

DR. SAMO thought we ought to vaccinate and revaccinate until it would no longer take. We could then, he believed, feel tolerably safe.

DR. BARTLETT makes it his routine practice, when vaccinating, to draw as little blood as possible. The humanized virus, in his estimation, if pure, is the best, while the bovine tends to become weakened by repeated transmissions. Dr. Bartlett has also noticed in his practice that the development of vaccinia is now different from what it was years ago. During the pustular stage of variola, he bathes the surfaces with a solution of bi-chloride, to abort the secondary fever.

DR. GROSVENOR uses a scarifier to vaccinate with. He has been in doubt laterally whether some of his vaccinations were successful. In the place of the usual vesicle and pustule, a strawberry-like mass appears.

DR. CALLAHAN called attention to the fact that emigrants' baggage could be brought here directly from Europe without fumigation, and suggested that all emigrants who stop in Buffalo with the intention of remaining should have their baggage subjected to a rigid disinfection. Dr. Callahan favored bovine virus.

DR. WYCKOFF was of the opinion that all virus is the same, and that it makes no difference whether we use humanized or bovine, so long as we are certain that the subject from whom the first was taken was healthy. When we use the scab or lymph from a successful vaccination, we are certain that the product is genuine, and not a spurious article coated on ivory points or quills and sold for purely mercenary objects. We should always have a sore if the virus is good and well inserted.

Dr. Pryor believed that the sale and propagation of virus should be under State control. It is so in England, and he thinks their virus is much superior to ours. Dr. Pryor exhibited some virus contained in fine glass tubes, which he had recently brought from England, and which, it was claimed, would last ten years without deterioration. He had already used some of the same with marked success.

Dr. Granger agreed with Dr. Pryor, and thought that the sale of virus at the existing prices should be discouraged, for it could not do otherwise than tend to cause the dealers to falsify their virus.

Dr. Coakley was of the opinion that bad results might follow the use of humanized virus, but never bovine. If the vaccination does not run through an approximately typical course, he believes that it is not successful. While in the army he scarified with a dull knife, and used a paste made from the scab. Care must be exercised not to draw too much blood.

Dr. Hartwig formerly scarified in six spots. From experiments made in Germany, it seems that at least five spots are necessary for absolute protection. He often wondered whether there ever was a disease, "cow-pox." Might it not have been in the beginning a case of small-pox communicated to the cow? Through his reading, he has been led to believe that this was Jenner's idea. Dr. Hartwig did not think it was necessary or right to move all small-pox cases at once to the hospital.

Dr. Clark believed that the humanized virus was the best, although it did not protect absolutely. Bovine virus varies; at present, that obtained from Dr. Martin's farm was giving the most satisfaction. In scarifying, we should draw no blood, and rub in well. He agreed with Dr. Callahan that the New York authorities were not careful enough in their inspection of emigrant baggage, and thought that the suggestion of local compulsory fumigation was good. Dr. Clark imagined that the present endemic had its origin in contagious material brought here by a Pole who came directly from an infected district in Europe. The case of Jackson, in its origin, was more obscure. A year ago, a solitary attack of variola was reported on Forest avenue. Jackson had been doing some whitewashing on Forest avenue shortly before he was taken sick, and Dr. Clark thought that he may have worked in the house where the case existed a year ago. Dr. Clark agreed with Dr. Pryor that the propagation and sale of virus ought to be under State control, and also with Dr. Hartwig that at each vaccination a number of scarifications should be made. The strawberry form following some scarifications is a spurious result in Dr. Clark's opinion.

Essayists for the October meeting announced as Dr. M. Hartwig: subject, "Chronic Pneumonia;" and Dr. J. H. Pryor: subject, "Results of Treatment by the Pneumatic Cabinet."

Adjourned at 10.15 P. M.

